# Supplementary material for: Crawling and Gliding: A Computational Model for Shape-Driven Cell Migration
Source: PLoS Comput Biol. 2015 Oct 21;11(10):e1004280. doi: 10.1371/journal.pcbi.1004280 (PMC4619082; doi:10.1371/journal.pcbi.1004280)
Supplement: S1 Code — (ZIP) [file pcbi.1004280.s012.zip › release/tst/doc/html/graph_8h_source.html]

Tissue Simulation Toolkit: graph.h Source File


Tissue Simulation Toolkit
 0.1.4.1


- Main Page
- Namespaces
- Classes
- Files

- File List
- File Members

graph.h

Go to the documentation of this file.

1 /\*

2

3 Copyright 1996-2006 Roeland Merks

4

5 This file is part of Tissue Simulation Toolkit.

6

7 Tissue Simulation Toolkit is free software; you can redistribute

8 it and/or modify it under the terms of the GNU General Public

9 License as published by the Free Software Foundation; either

10 version 2 of the License, or (at your option) any later version.

11

12 Tissue Simulation Toolkit is distributed in the hope that it will

13 be useful, but WITHOUT ANY WARRANTY; without even the implied

14 warranty of MERCHANTABILITY or FITNESS FOR A PARTICULAR PURPOSE.

15 See the GNU General Public License for more details.

16

17 You should have received a copy of the GNU General Public License

18 along with Tissue Simulation Toolkit; if not, write to the Free

19 Software Foundation, Inc., 51 Franklin St, Fifth Floor, Boston, MA

20 02110-1301 USA

21

22 \*/

23

31 #ifndef \_GRAPH\_H\_

32 #define \_GRAPH\_H\_

33 #include <iostream>

34 // Base class for Graphics interface. No implementation

35

36 class Graphics {

37

38  public:

39  // Graphics(int xfield, int yfield, const char \*movie\_file=0);

40  virtual ~Graphics(void) {};

41

43  virtual void BeginScene(void) {

44  };

46  virtual void EndScene(void) {

47  };

53  virtual void Point( int color, int x, int y)=0;

54

61  virtual void Line(int x1, int y1,int x2,int y2,int colour )=0;

62

70  virtual int GetXYCoo(int \*X,int \*Y)=0;// {return 0;}

71

73  virtual int XField(void) const {return 0;}

74

76  virtual int YField(void) const {return 0;}

77

87  virtual void Write(char \*fname, int quality=-1)=0;

88

94  virtual void TimeStep(void) {};

95

105  virtual void Field(const int \*\*f, int mag=1) {

106  throw "Graphics::Field not implemented. Try X11 graphics.\n";

107  }

108

109 };

110

111

112 #endif

113

Graphics::Field

virtual void Field(const int \*\*f, int mag=1)

Plots a field of values given by \*\*f, using color coding given by colormap file.

**Definition:** graph.h:105

Graphics::XField

virtual int XField(void) const

Returns the width of the Graphics window, in pixels.

**Definition:** graph.h:73

Graphics::YField

virtual int YField(void) const

Returns the height of the Graphics window, in pixels.

**Definition:** graph.h:76

Graphics::TimeStep

virtual void TimeStep(void)

Implement this member function in your simulation code.

**Definition:** graph.h:94

Graphics::Point

virtual void Point(int color, int x, int y)=0

Plot a point in the Graphics window.

Graphics::Line

virtual void Line(int x1, int y1, int x2, int y2, int colour)=0

Draws a line (obviously... :-)

Graphics::GetXYCoo

virtual int GetXYCoo(int \*X, int \*Y)=0

Probes the Window for user interaction, with mouse or keyboard.

Graphics::Write

virtual void Write(char \*fname, int quality=-1)=0

Writes the Image to a file.

Graphics

API for Graphics windows.

**Definition:** graph.h:36

Graphics::EndScene

virtual void EndScene(void)

EndScene() must be called to flush the drawing buffer and display the scene.

**Definition:** graph.h:46

Graphics::~Graphics

virtual ~Graphics(void)

**Definition:** graph.h:40

Graphics::BeginScene

virtual void BeginScene(void)

BeginScene() must be called before calling drawing functions.

**Definition:** graph.h:43


---

Generated on Thu Aug 14 2014 22:04:01 for Tissue Simulation Toolkit by  

 1.8.6
